# Azithromycin as a Possible Cause of Linear IgA Bullous Dermatosis

**DOI:** 10.7759/cureus.38592

**Published:** 2023-05-05

**Authors:** Cailin O’Connell, Nicole N Dacy, Shannon C Brown, Lisa Lopez

**Affiliations:** 1 Engineering Medicine, Texas A&M School of Medicine, Houston, USA; 2 Department of Dermatology, Baylor Scott & White Health, Temple, USA; 3 Department of Pathology, Baylor Scott & White Health, Temple, USA

**Keywords:** bpag2, immunobullous disorder, azithromycin, drug-induced, linear iga bullous dermatosis

## Abstract

We present a rare case of linear IgA bullous dermatosis (LABD) in a 72-year-old male associated with the use of azithromycin. LABD presents as subepidermal blisters due to IgA antibodies targeting BPAG2, a component of hemidesmosomes. LABD is a rare diagnosis and may be idiopathic, associated with illness, or medication-induced. The patient experienced a rash five days after completing a course of azithromycin for pneumonia. The diagnosis of LABD was confirmed with a biopsy and direct immunofluorescence. Lesions resolved over two weeks with an oral prednisone taper and topical clobetasol. This case represents just one of two previously reported cases in the literature of azithromycin-associated LABD. While LABD is well known to be induced by certain medications, this is only the second report of it being associated with the use of a macrolide. We propose that macrolides be included as a potential cause of medication-induced LABD.

## Introduction

Linear IgA bullous dermatosis (LABD) is an immune-mediated, vesiculobullous disease with a bimodal distribution, typically occurring in early childhood or after age 60 [1]. LABD is relatively rare and has an incidence of 0.2 to 2.3 cases per million people [2]. LABD pathogenesis involves the production of IgA antibodies against BPAG2 antigen components of hemidesmosomes, leading to the formation of subepidermal blisters [3]. Classically, LABD presents as widespread, tense vesicles or bullae in an arcuate or annular pattern. Lesions typically appear on the trunk, extremities, buttocks, and perioral region. The differential includes other immunobullous disorders such as bullous pemphigoid and dermatitis herpetiformis [4]. LABD may be idiopathic or triggered by autoimmune conditions, malignancies, infections, or medications. Below, we discuss a case of newly diagnosed LABD in a 72-year-old male following the use of azithromycin. 

## Case presentation

**Figure 1:**
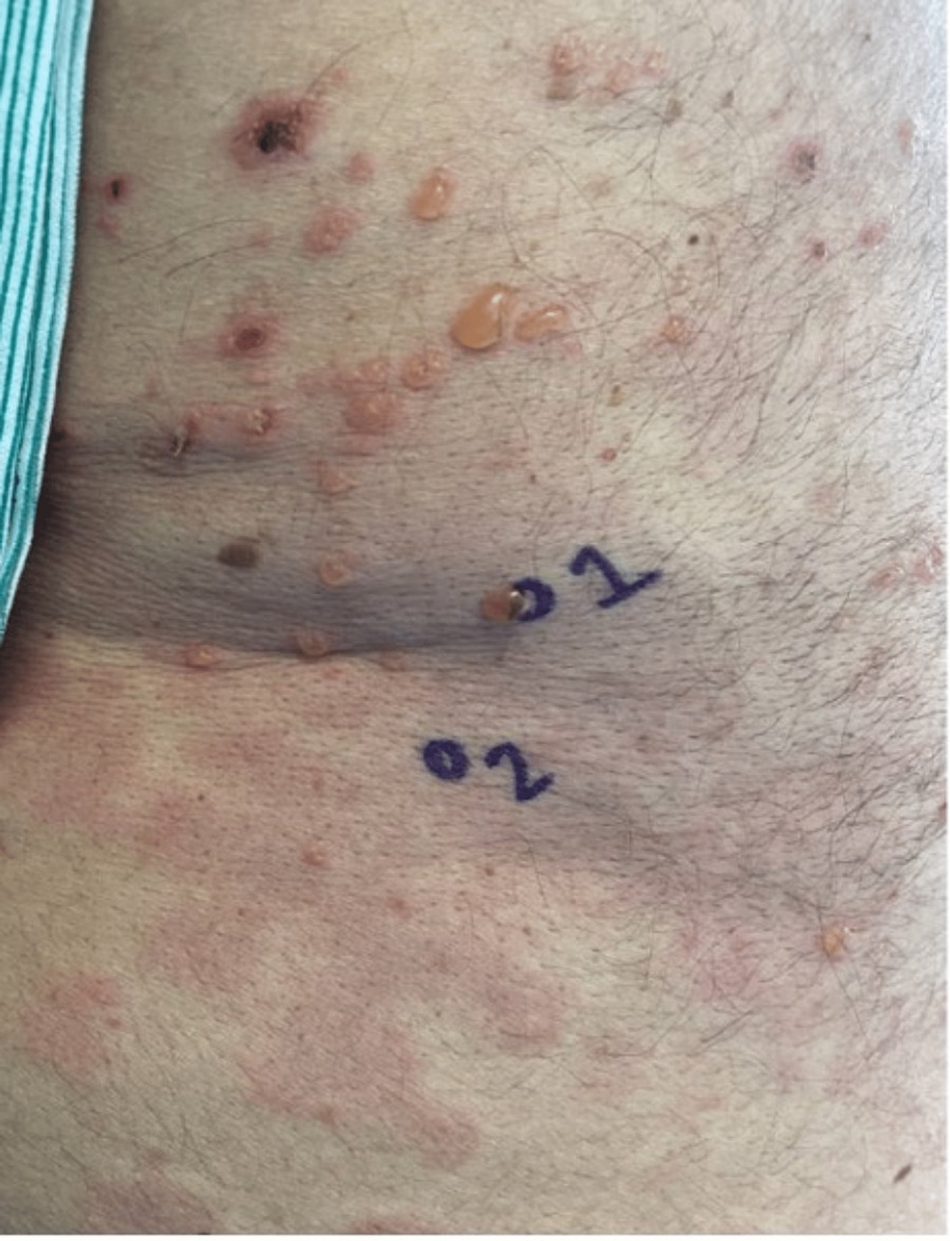
Left flank showing tense bullae on erythematous background. Biopsy site 1 was stained with H&E, and biopsy site 2 was used for DIF. H&E: hematoxylin and eosin; DIF: direct immunofluorescence

A 72-year-old male presented to the emergency department with a cough and a two-day history of a worsening rash. Twelve days prior to presentation, he was diagnosed with pneumonia and prescribed a five-day course of azithromycin along with a nine-day oral prednisone taper. Past medical history was significant for hypertension, hyperlipidemia, chronic kidney disease, and type 2 diabetes mellitus. The patient had no recent travel or animal exposures. A physical exam demonstrated scattered tense bullae and vesicles overlying an erythematous background on the upper extremities, back, and chest (Figure 1). No mucosal involvement was noted. A complete blood count was significant for an elevated white blood cell count (18,200/mm^3^) with a peripheral blood smear demonstrating absolute neutrophilia, and a comprehensive metabolic panel was within normal limits. The respiratory virus panel and COVID-19 PCR were negative. A CT scan of the chest showed nonspecific mediastinal and hilar lymphadenopathy.

**Figure 2:**
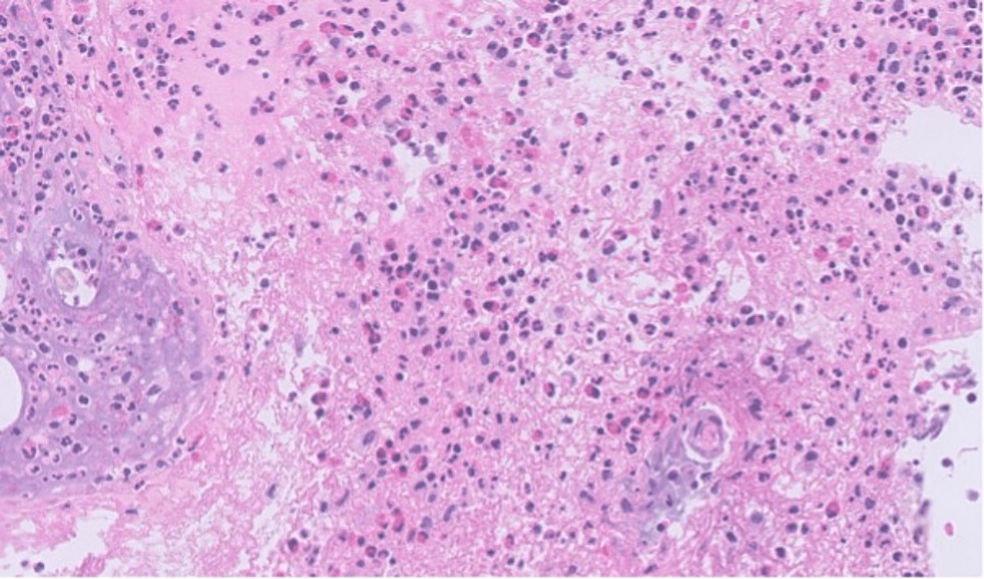
Punch biopsy H&E staining demonstrating subepidermal bulla with fibrin and mixed inflammation consisting of neutrophils and eosinophils. H&E: hematoxylin and eosin

**Figure 3:**
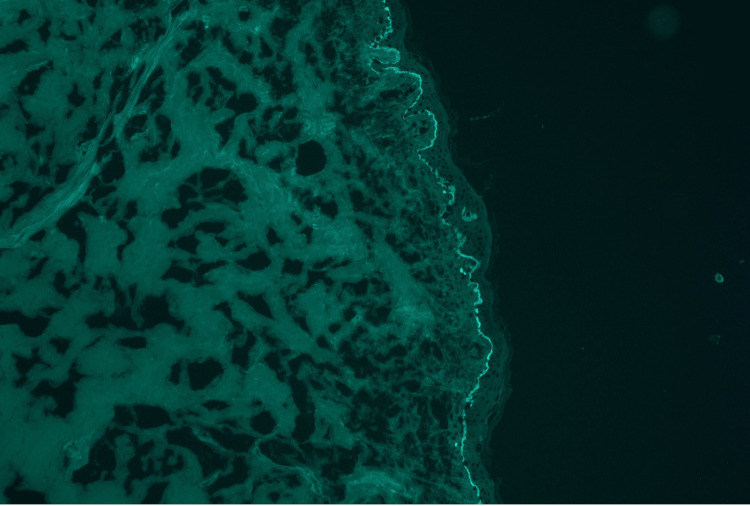
Direct immunofluorescence of punch biopsy revealing linear staining of IgA at the dermal-epidermal junction, consistent with linear IgA bullous dermatosis.

**Figure 4:**
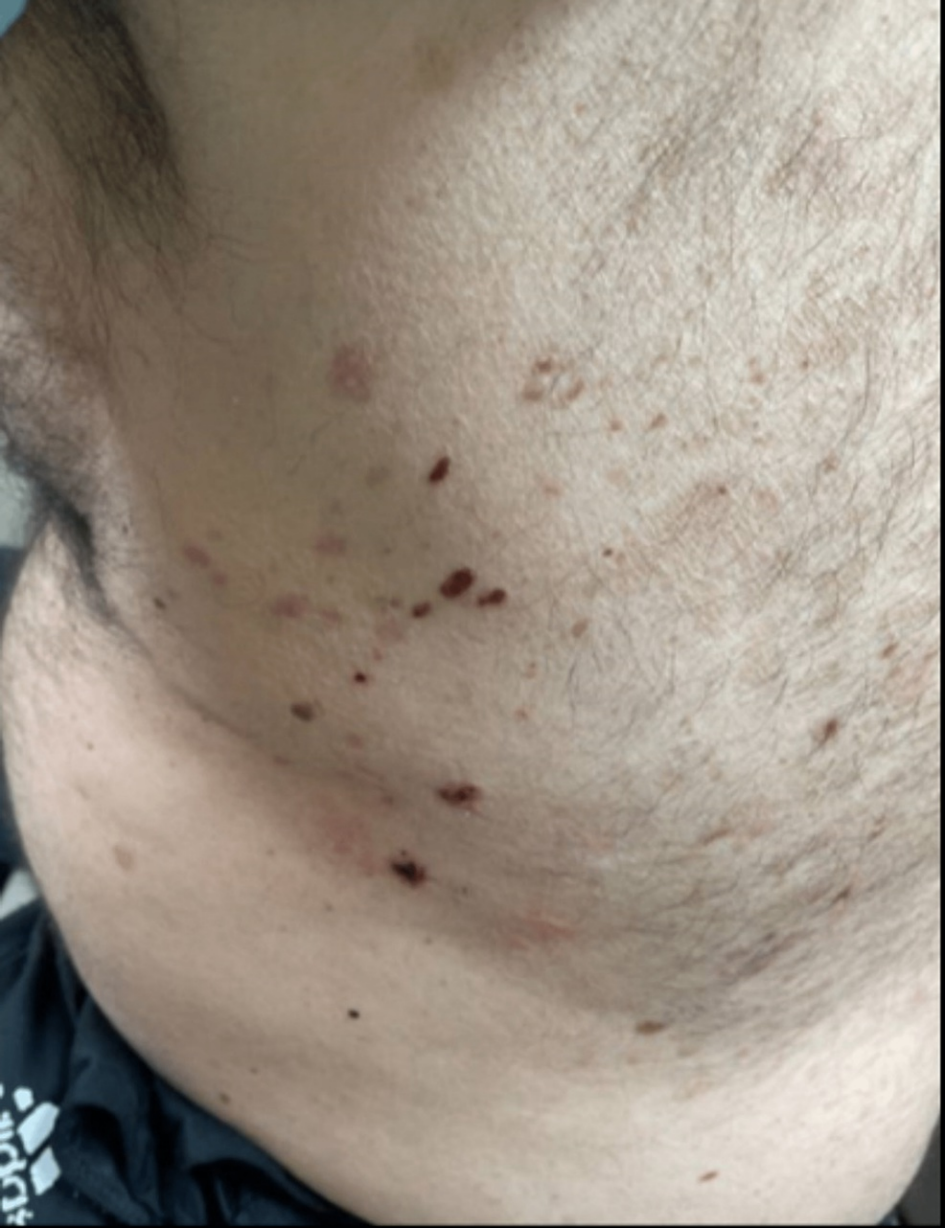
Resolution of trunk erythematous base and bullae at the clinic follow-up visit.

Histology showed a subepidermal bulla with fibrin and mixed inflammation consisting of neutrophils and eosinophils (Figure 2). Direct immunofluorescence (DIF) revealed 1-2+ linear IgA staining along the dermal-epidermal junction (DEJ) (Figure 3). Serum bullous pemphigoid-180 and 230 IgG antibodies were negative. These findings were consistent with a diagnosis of LABD. Given his recent history of azithromycin use, negative respiratory viral panel, and lack of other recent medication changes, it was felt azithromycin was the most likely trigger. After stabilization of respiratory status, the patient was discharged with a three-week prednisone taper (60 mg for one week, 40 mg for one week, and 20 mg for one week) and topical clobetasol ointment (0.05%). The lesions resolved within two weeks without the need for further treatment (Figure 4). The patient was advised to avoid azithromycin exposure in the future.

## Discussion

LABD is a rare immune-mediated bullous disease that can be idiopathic, associated with malignancy, or induced by infection or medications. Typical clinical presentation is vesiculobullous lesions with or without erythema across the trunk, extremities, and buttocks, and may also have membranous involvement [2]. The known pathophysiology of the disease involves IgA antibodies that are generated against two BPAG2 related antigens: LAD-1 (120 kD cleaved portion of the BP180 antigen) and LABD97 (97 kD cleaved portion of LAD-1) [5]. BPAG2 is a component of hemidesmosomes, which are responsible for maintaining the DEJ in the lamina lucida [3]. Disruption of BPAG2 by IgA antibodies leads to the classic histopathologic findings of LABD. These findings include a subepidermal blister with neutrophils, DIF revealing linear IgA along the basement membrane, and indirect immunofluorescence of salt-split skin with staining of the epidermal side or “roof” of the blister [6,7]. Increased IL-5 expression has also been implicated in the pathophysiology of drug-induced LABD (DI-LABD), a phenomenon that was reported in cases of DI-LABD caused by ceftriaxone and metronidazole [8]. The first-line treatment for LABD is dapsone. If patients are unable to tolerate dapsone, sulfapyridine can be used. Topical corticosteroids are also employed for mild cases, and in patients with suspected DI-LABD, identification and cessation of the offending medication is vital [9]. After discontinuing the associated medication, termination of new lesion formation can be observed in days or weeks. Idiopathic cases may persist for years following the initial presentation [10,11].

The most common medication reported to cause DI-LABD is vancomycin. Less common culprits include penicillins, cephalosporins, captopril, and nonsteroidal anti-inflammatory drugs [12]. DI-LABD typically presents one to 14 days after initiation of inciting medication [10].

To our knowledge, there has only been one other report of a possible macrolide-associated DI-LABD. Cummings et al. described the case of a 54-year-old man who developed Stevens-Johnson like DI-LABD after being treated with azithromycin, rimantadine, and zanamivir for presumed influenza [13]. Similar to our patient, this patient’s lesions resolved with a course of oral prednisone and pentoxifylline and discontinuation of the three possible offending agents. Interestingly, our patient did not develop lesions until five days after finishing his course of azithromycin. We believe that his concomitant short burst of prednisone suppressed IL-5 and antibody formation, thus attenuating his clinical presentation until the prednisone dose was decreased. He responded quickly once the dose of prednisone was increased again.

## Conclusions

We presented the case of a 72-year-old male with biopsy-proven LABD in association with recent azithromycin use with disease resolution after medication cessation, an increased dose of oral prednisone, and topical clobetasol. We propose that macrolides, specifically azithromycin, be included as potential causes of drug-induced LABD. Patients may improve with cessation of the offending medication alone or may require treatment with systemic steroids.
